# Potential role of endoplasmic reticulum stress is involved in the protection of fish oil on neonatal rats with necrotizing enterocolitis

**DOI:** 10.1038/s41598-020-63309-9

**Published:** 2020-04-15

**Authors:** Xiaoli Zhu, Ningxun Cui, Lingling Yu, Ping Cheng, Mingling Cui, Xueping Zhu, Jian Wang

**Affiliations:** 1 Department of Intervention, The First Affiliated Hospital of Soochow University, Suzhou, 215006 China; 2 Department of Neonatology, Children’s Hospital of Soochow University, Suzhou, 215025 China; 3 Department of Neonatology Surgery, Children’s Hospital of Soochow University, Suzhou, 215025 Jiangsu China

**Keywords:** Immunization, Inflammatory bowel disease

## Abstract

Neonatal necrotizing enterocolitis (NEC) is a serious gastrointestinal disease with high death rate in premature infants. Fish oil (FO) and its constituents have been shown to ameliorate intestinal inflammation and mucosal damage. However, the underlying mechanism of action is not known. In the present study, we divided Sprague-Dawley rats into three groups: control group, NEC model group, and FO pre-feeding+NEC model group. Briefly, one week before NEC modeling, in addition to being fed with milk, the FO pre-feeding+NEC modeling group was fed with FO, the NEC group was fed with saline, and the control group was only inserted a gastric-tube for 7 days. Subsequently, histological assay, Western blot, and ELISA were performed. Pretreatment with FO attenuated the NEC symptoms, alleviated intestinal pathological injury, and decreased the expressions of pro-inflammatory cytokines such as interleukin-6 (IL-6) and tumor necrosis factor-α (TNF-α). Furthermore, pretreatment with FO reduced the expressions of endoplasmic reticulum stress (ERS) related proteins, caspase-12, and glucose-regulated protein 78 (GRP78). In addition, intestinal histopathological scores showed a significant positive correlation with intestinal expressions of IL-6, TNF-α, and caspase-12. Collectively, these results indicate that ERS pathway might be involved in the effect of FO in alleviating intestinal mucosal inflammation and injury in rats with NEC

## Introduction

Neonatal necrotizing enterocolitis (NEC) is a serious gastrointestinal disease that affects premature infants and is associated with a high mortality rate. Various factors such as premature birth, formula feeding, intestinal ischemia, and bacterial colonization are known to activate the intestinal inflammatory response that ultimately induces NEC^1^. A variety of inflammatory factors such as platelet activating factor (PAF), tumor necrosis factor alpha (TNF-α), interleukin (IL)-6, and IL-8 have been implicated in the pathogenesis of NEC^2^. Elevated levels of IL-6 and IL-8 were found in infants with NEC^3^. During the process of NEC, TNF-α was shown to induce significant mitochondrial dysfunction and mitochondrial apoptosis, which eventually led to apoptosis of intestinal epithelial cells^4^. Recently, the anti-inflammatory effects of docosahexaenoic acid (DHA) and arachidonic acid were shown to prevent NEC in premature infants^5^. Insulin-like growth factor I^6^ and berberine^7^ were shown to attenuate inflammation and ameliorate the clinical symptoms and progression of NEC. Taken together, the mechanism for alleviating NEC effectively is worthy of study.

The endoplasmic reticulum (ER) is an organelle with characteristic flattened, membrane-enclosed sacs or tube-like structures. ER includes rough ER and smooth ER. Rough ER is studded with ribosomes and plays a role in protein synthesis, while the smooth ER is involved in lipid synthesis and metabolism. Harmful stimuli such as hypoxia, infection, oxidative stress, and harmful metabolites disrupt homeostasis in ER and cause ER dysfunction which further induces endoplasmic reticulum stress (ERS). ERS triggers the expressions of unfolded protein response (UPR) and glucose-regulated protein 78 (GRP78) in the cytoplasm. Hence, GRP78 is a classical marker of ERS^8^. In addition, caspase-12, which is located on the cytoplasmic side of the ER, was shown to eliminate damaged cells that could not be repaired in time; this process was mediated via an ER-specific apoptosis pathway^9^. Failure of UPR to restore ER homeostasis leads to cellular apoptosis and local inflammation. Lu *et al*.^10^. demonstrated that ERS and UPR in NEC were associated with increased levels of IL-6 and IL-8 along with severe epithelial injury. Therefore, the relationship between ERS and NEC needs to be further clarified.

Fish Oil (FO) is rich in DHA, eicosapentaenoic acid (EPA), and ω-3 polyunsaturated fatty acids (ω-3 PUFAs). ω-3 PUFAs in FO were shown to ameliorate inflammation and mucosal damage in ulcerative colitis^11^. In addition, it was shown to attenuate intestinal inflammation and reduce the incidence of NEC in neonatal rats^12^. It was also shown to prevent ERS-mediated apoptosis of primary rat hepatocytes^13^. The anti-inflammatory effects of DHA are mediated via modulation of ER homeostasis^14^. DHA was shown to protect astrocytes against ischemic injury^15^ and muscle cells against palmitate-induced atrophy via inhibition of ERS^16^. EPA enhanced the integrity of the intestinal mucosal epithelial barrier via up-regulation of the expression of tight junction proteins in a rat model of heatstroke^17^. In addition, it was shown to attenuate statin-induced ERS in cultured myoblast cells^18^. These findings indicate the protective effect of FO in intestinal cells. However, further studies are required to unravel the underlying mechanism of the protective effect.

In the present study, we investigated the ERS-coupled inflammatory response by determining the expressions of ERS-related marker proteins and pro-inflammatory cytokines in intestinal tissue of neonatal rats with NEC. We further evaluated the repressive effect of FO on ERS-coupled intestinal inflammation with an aim to uncover the therapeutic potential of FO against NEC.

## Materials and methods

### Animals and experimental design

A total of 96 one-day-old neonatal Sprague-Dawley (SD) rats (clean grade, body weight: 5–10 g) were randomly divided into three equal groups (control, NEC, and FO pre-feeding +NEC groups, n = 32). Rats in the control group were fed with milk throughout the experiment, and were inserted with gastric tube once a day for 7 days; subsequently, they were administered an equal amount of saline by gavage once a day at 12:00 Hrs for three days. In addition to being fed with milk, rats in the NEC group were administered saline by gavage for seven days till the day of performing NEC modeling with “formula feeding + hypoxia + cold stimulation + LPS gavage” combined multi-factor method for three days. In addition to milk, rats in the FO pre-feeding +NEC group were pre-fed with FO (contains 35% of DHA and EPA totally, 0.6 mL/100 g/d) (Sigma, St. Louis, MO, USA) by gavage for seven days (based on our preliminary experiment) till the day of performing NEC modeling. All rats in each group were housed in a single cage at a temperature of 25 °C–30 °C and 50–60% humidity with a 12 h light: 12 h dark cycle. During the experiments, the behavior of animals and the state of their feces were recorded. NEC modeling was performed according to the protocols published by Hunter *et al*.^19^ and Welak *et al*.^20^ Briefly, all rats were administered 0.4 mL formula milk four times (08:00 Hrs, 12:00 Hrs, 16:00 Hrs, 20:00 Hrs) a day by gavage. At 08:00 Hrs and 20:00 Hrs, rats in the NEC group and FO pre-feeding +NEC group were placed in hypoxic chambers (nitrogen flow: 15 L/min) twice a day for three days. The rats were placed in the hypoxic chambers for 90 s when the oxygen concentration in the chamber was down to 0, and subsequently removed to 4 °C for 10 mins. At 12:00 Hrs, rats in the NEC group and FO pre-feeding +NEC group were administered 10 mg/kg BW lipopolysaccharide (LPS, Sigma, St. Louis, MO, USA) by gavage at 12:00 Hrs every day for three days.

At 0 h, 24 h, 48 h, and 72 h of NEC modeling, eight rats in each group were randomly sacrificed and their intestinal samples were collected. This study was carried out in accordance with the *Guiding Principles for the Care and Use of Laboratory Animals of the Soochow University*. Animals were treated in accordance with Guide for the Care and Use of Laboratory Animals (8th edition, National Academies Press). The experimental protocols were approved by the *Ethics Committee for Animal Experiments at the Soochow University* (NO.2013LW003).

### Histological assay

Intestinal tissues (2 cm proximal to the ileocecal region) were collected and fixed in 4% paraformaldehyde. Paraffin sections (thickness: 5 μm) were prepared for hematoxylin & eosin (HE) staining prior to histopathological examination. The intestinal histopathological damage was scored by a single-blinded evaluator according to a standard described by Nadler *et al*.^21^ (Table 1). Three random fields (40×) from each sample were evaluated and the rats were diagnosed as NEC when the score was greater than two.

**Table 1:** Standard criteria for intestinal histopathological scoring.

Score	Histological morphology
0	Intestinal villus and mucosa epithelium are intact
1	Slight separation between the sub-mucosa and the lamina propria
2	Moderate separation between the sub-mucosa and the lamina propria OR sub-mucosal edema OR muscular layer edema
3	Marked separation between the sub-mucosa and lamina propria OR severe sub-mucosal edema OR severe muscular layer edema OR villous fall off
4	Disappearance of intestinal villi OR intestinal villus necrosis

For immunostaining, the intestinal cross sections were incubated overnight with caspase-12 (SC-21747, Santa Cruz Biotechnology Inc, Dallas, TX, USA) or GRP78 (MB0050, Bioworld, USA) primary antibody at 4 °C. Subsequently, biotin anti-rabbit IgG and 3,3’-diaminobenzidine (DAB) were used for visualization. The results were expressed as gray value analysis in five random fields (40×) from each section by using Image-Pro-Plus 5.0 software.

### Western Blot

The total protein from intestinal samples (0.1 g) was extracted by lysis using 0.3 mL RIPA buffer (Beyotime, Jiangsu, China) with 1% PMSF (ST506, Beyotime, Jiangsu, China). The total protein was separated by SDS-PAGE, followed by electro-transfer to a polyvinylidene fluoride membrane (PVDF, Millipore, Billerica, MA, USA). The PVDF membrane was incubated overnight with caspase-12 primary antibody (1:1000) at 4 °C. After incubation with secondary antibody (1:3000) for 1 h at 37 °C, the membranes were visualized using an ECL system (Bio Rad Laboratones, Inc., Hercules, CA, USA). The amount of protein was determined using the Image J software. The data were expressed as the gray value of the bands normalized to the gray value of the corresponding β-actin bands. Each experiment was performed in triplicate.

### ELISA

The intestinal tissue was homogenized in ice-cold physiological saline (PBS). The supernatant was harvested following centrifugation at 5000 rpm for 15 min at 4 °C and was stored at −80 °C. The levels of intestinal cytokines IL-6 and TNF-α were determined using a commercial ELISA kit (R&D system, US) according to the manufacturer’s instructions. The OD values were determined at a wavelength of 450 nm. Standard curve was used to calculate the concentrations of IL-6 and TNF-α. Each sample was analyzed in triplicate

### Data analysis

Data are presented as mean ± standard error of the mean (SEM). Differences between various groups were assessed using one-way ANOVA. SPSS 20.0 (SPSS Inc, Chicago, IL, USA) was used for statistical analyses. Least significant difference (LSD) post hoc multiple comparisons test was used to determine the statistical differences. P values <0.05 were considered indicative of statistical significance.

### Ethical approval and informed consent

1. Approval: The experimental protocols were approved by the Ethics Committee for Animal Experiments at the Soochow University (NO.2013LW003).

2. Accordance: The methods were carried out in accordance with the relevant guidelines and regulations.

## Results

### FO attenuates intestinal mucosal injury in rats with NEC

**Figure 1:**
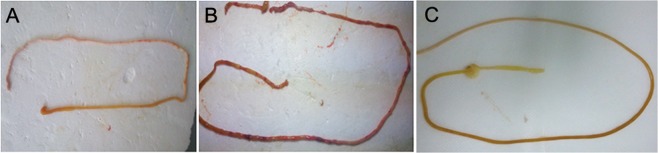
Necropsy findings of rats with NEC. (**A**) Intestinal morphology in the control group shows no signs of obvious intestinal luminal flatulence. (**B**) At 72 h of NEC modeling, the small intestine in NEC group showed black appearance with signs of luminal flatulence. (**C**) At 72 h of NEC modeling, the small intestine in the FO + NEC group exhibited deep yellow appearance with no obvious signs of intestinal luminal flatulence.

**Figure 2:**
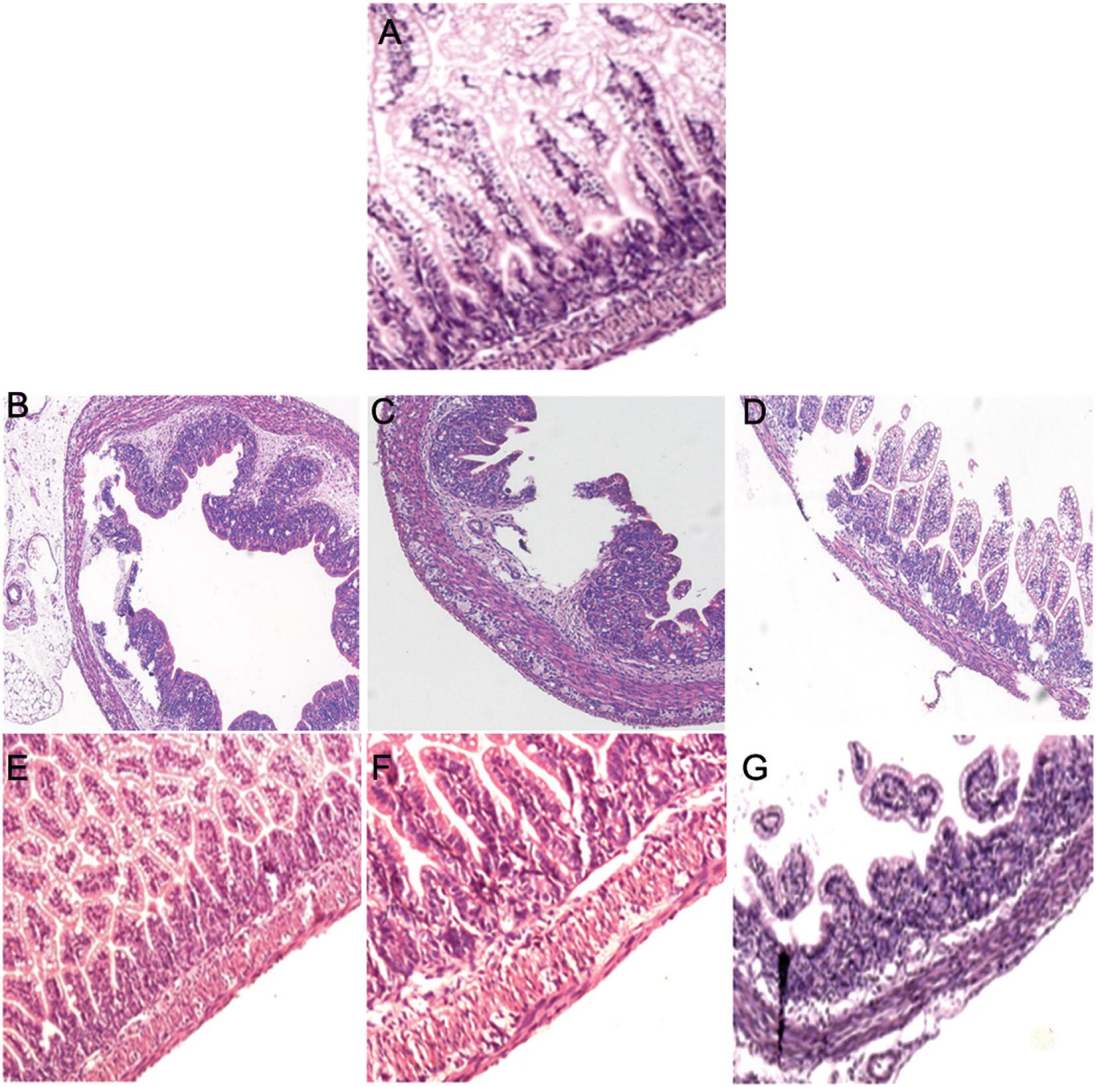
Histopathological analysis of intestine of rats with NEC. (**A**) Intestinal tissue of the control group showing intact intestinal villi and mucosal epithelium. (**B–D**) At 24 h, 48 h, and 72 h of NEC modeling, the intestinal mucosa in NEC group exhibited separation between the sub-mucosa and the lamina propria, intestinal villus fall off, and mucosal neutrophil infiltration. (**E–G**) At 24 h, 48 h, and 72 h of NEC modeling, FO pre-feeding attenuated NEC-induced intestinal mucosal pathological damage.

In contrast to the control group, rats in the NEC group developed symptoms such as abdominal distension, diarrhea, respiratory distress, and watery stools. At necropsy performed at 72 h of NEC modeling (Fig. 1), the intestines of rats in the NEC group showed black appearance with signs of luminal flatulence. Histopathological analysis (Fig. 2, Table 2) revealed gradual aggravation of intestinal mucosal injury with progression of NEC modeling. At 72 h of NEC modeling, most of the intestinal villi in the NEC group exhibited disordered arrangement, fall off, or necrosis. Further, separation between sub-mucosa and lamina propria, extensive neutrophil infiltration in the mucosa, and sub-mucosal edema were observed. Moreover, the pathological scores of NEC group were significantly elevated during NEC progression. Symptoms in the FO + NEC group (such as diarrhea, respiratory distress, watery stool, and luminal flatulence) were much milder than those in the NEC group. At 24 h, 48 h and 72 h, the intestinal pathological scores in the FO + NEC group were lower than those in the NEC group by 38.79%, 27.49%, and 36.20%, respectively (P < 0.05 at all 3 time-points).

**Table 2:** Intestinal histopathological scores of rats in various study groups (n = 8).

Group	0 h	24 h	48 h	72 h
Control	0.04 ± 0.12	0.08 ± 0.15	0.08 ± 0.15	0.12 ± 0.17
NEC	0.04 ± 0.12	1.16 ± 0.31^*Δ^	1.71 ± 0.45^*Δ^	2.79 ± 0.66^*Δ^
FO + NEC	0.04 ± 0.12	0.71 ± 0.28^*#Δ^	1.24 ± 0.34^*#Δ^	1.78 ± 0.56^*#Δ^

^*^P < 0.05 versus control group; ^#^*P* < 0.05 versus NEC group; ^△^*P* < 0.05 versus the immediately preceding time –point.

### FO attenuated intestinal mucosal inflammation in rats with NEC

Consistently lower expressions of intestinal IL-6 and TNF-α were observed in the control group (Tables 3 and 4). During NEC modeling, the expression levels of IL-6 and TNF-α showed a gradual increase over time. The IL-6 levels in the NEC group at 24, 48, and 72 h were higher than the respective levels in the control group by 7.98%, 51.89%, and 71.90%, respectively (P < 0.05 at all 3 time-points). Similarly, the TNF-α level in the NEC group were higher than those in the control group by 4.59%, 33.98%, and 69.16%, respectively. High expression levels of IL-6 and TNF-α were observed in the FO + NEC group at 24 h, 48 h and 72 h; however, the IL-6 levels in the FO + NEC group at 24, 48, and 72 h were lower than those in the NEC group by 4.30%, 9.40%, and 13.70%, respectively (P < 0.05), while the TNF-α levels in FO + NEC group were lower than those in the NEC group by 3.07%, 8.26%, and 11.37%, respectively.

**Table 3:** Rat intestinal IL-6 expression in various study groups (pg/mL, n = 8).

Group	0 h	24 h	48 h	72 h
Control	146.34 ± 8.49	149.55 ± 12.59	151.21 ± 11.69	155.87 ± 12.01
NEC	147.26 ± 10.81	161.49 ± 12.57^Δ^	229.68 ± 13.64^*Δ^	267.94 ± 20.94^*Δ^
FO + NEC	150.05 ± 9.39	154.54 ± 10.50	208.09 ± 15.42^*#Δ^	231.24 ± 18.16^*#Δ^

^*^P < 0.05 versus control group; #*P* < 0.05 versus NEC group; ^△^*P* < 0.05 versus the immediately preceding time –point.

**Table 4:** Rat intestinal TNF-α expression in various study groups (pg/mL, n = 8).

Group	0 h	24 h	48 h	72 h
Control	146.32 ± 7.57	149.11 ± 8.73	149.40 ± 9.78	151.04 ± 10.12
NEC	146.10 ± 8.75	155.95 ± 9.24^Δ^	200.16 ± 12.97^*Δ^	255.50 ± 16.05^*Δ^
FO + NEC	145.74 ± 9.12	151.17 ± 10.43	183.63 ± 13.78^*#Δ^	226.46 ± 15.01^*#Δ^

^*^P < 0.05 versus control group; ^#^*P* < 0.05 versus NEC group; ^△^*P* < 0.05 versus the immediately preceding time –point.

### Effect of FO on intestinal caspase-12 protein expression in rats with NEC

**Figure 3:**
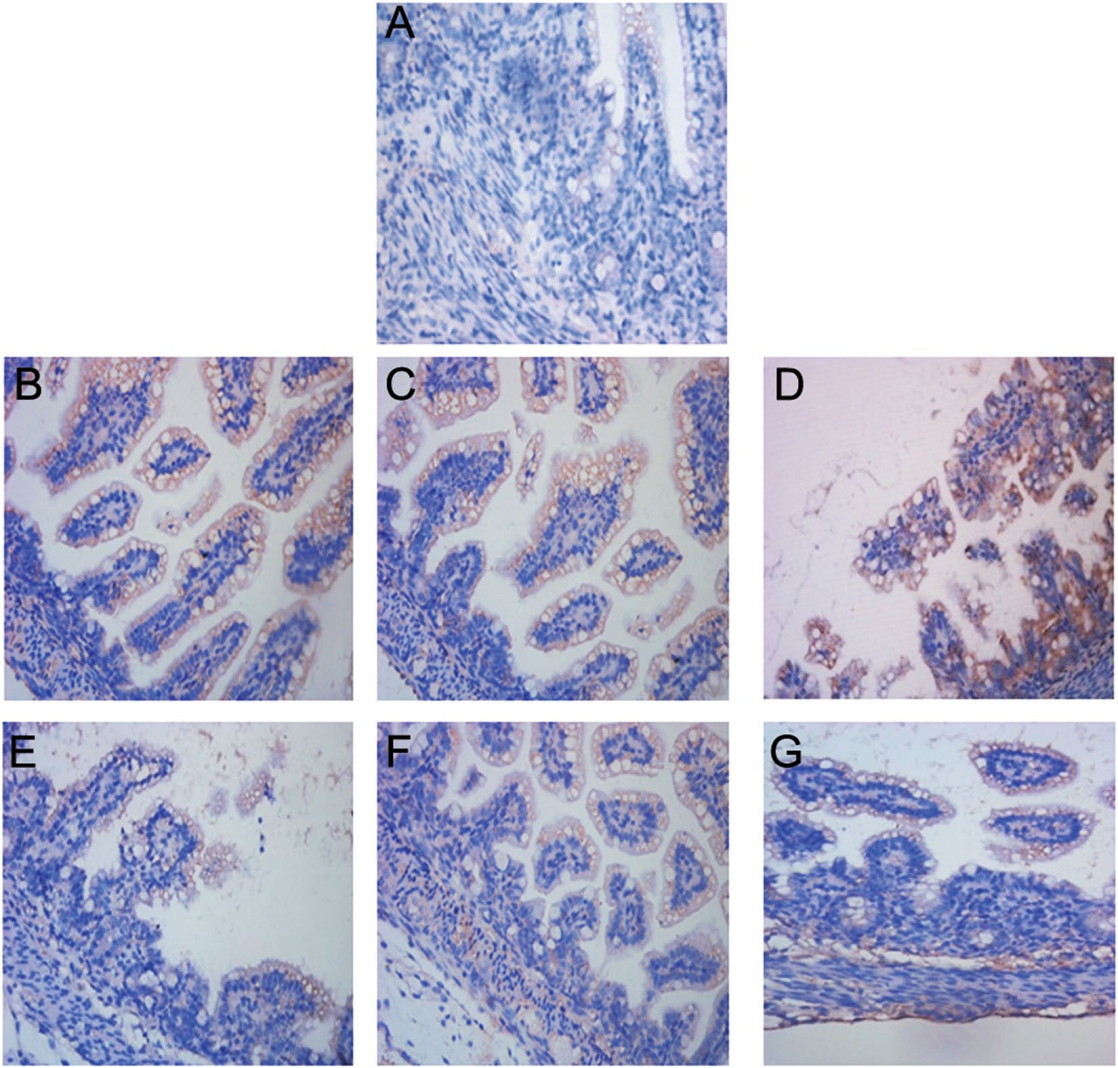
Expression analysis of caspase-12 in intestine of rats with NEC. (**A**) Immunostaining for intestinal caspase-12 protein in the control group showing caspase-12-positive cells mainly located in the intestinal epithelial cells and the sub-mucosa. (**B–D**) At 24 h, 48 h, and 72 h of NEC modeling, the Caspase-12-positive cells in the NEC group are mainly located around the intestinal epithelial cells, mucosa and the sub-mucosa. (**E–G**) At 24 h, 48 h, and 72 h of NEC modeling, the expression of intestinal caspase-12 protein in the intestinal mucosa in the FO + NEC group is markedly reduced.

**Figure 4:**
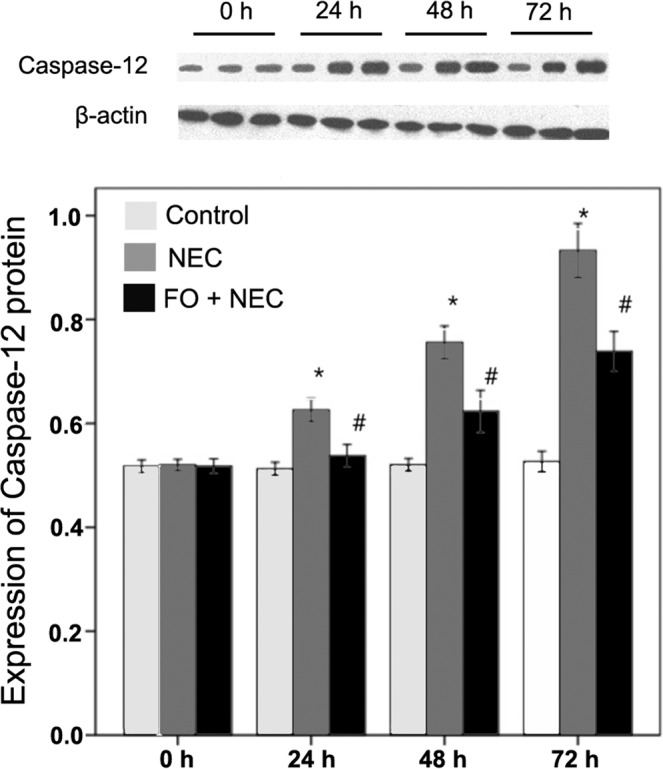
Quantitative analysis of intestinal caspase-12 protein by Western blot. The histogram represents the ratio of optical density of caspase-12 to that of β-actin. *P < 0.05 for NEC versus control group; #P < 0.05 for FO + NEC versus NEC group.

In the present study, caspase-12-positive cells exhibited yellow-brown staining in the cytoplasm, and were mainly distributed around the intestinal mucosal epithelial cells and in the sub-mucosa (Fig. 3). The results of Western blotting showed that the protein expressions of caspase-12 in the NEC group at 24 h, 48 h and 72 h of NEC modeling were greater than those in the control group by 22.02%, 45.25%, and 77.12%, respectively (*P* < 0.05 at all 3 time-points) (Fig. 4, Table 5). After pretreatment with FO, the expressions of caspase-12 at 24 h, 48 h, and 72 h of NEC modeling were greater than those in the control group by 6.61%–40.28%, but were lower than those in the NEC group by 12.63%, 17.57%, and 20.80%, respectively (P < 0.05 at all 3 time-points).

**Table 5:** Gray value analysis of intestinal caspase-12 protein expression in rats (n = 8).

Group	0 h	24 h	48 h	72 h
Control	0.5176 ± 0.0146	0.5126 ± 0.0120	0.5204 ± 0.0146	0.5266 ± 0.0237
NEC	0.5198 ± 0.0131	0.6255 ± 0.0266^*Δ^	0.7559 ± 0.0379^*Δ^	0.9327 ± 0.0622^*Δ^
FO + NEC	0.5177 ± 0.0171	0.5465 ± 0.0315^*#Δ^	0.6231 ± 0.0487^*#Δ^	0.7387 ± 0.0458^*#Δ^

^*^P < 0.05 versus control group; ^#^*P* < 0.05 versus NEC group; ^△^*P* < 0.05versus the immediately preceding time -point.

### Effect of FO on intestinal GRP78 protein expression in rats with NEC

**Figure 5:**
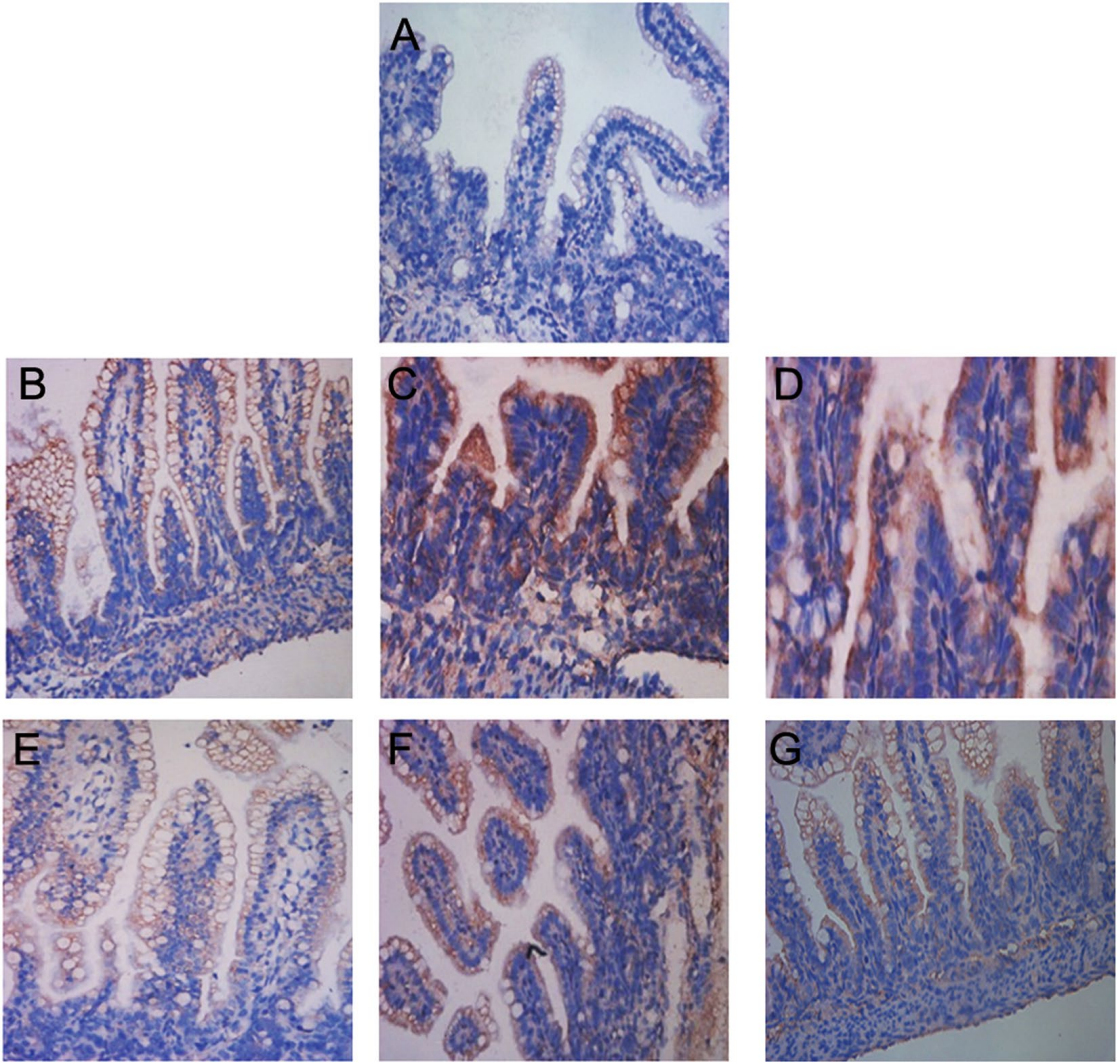
Expression of GRP78 in intestine of rats with NEC. (**A**) The expression of GRP78 protein in the control group; GRP78-positive cells are mainly distributed in the intestinal epithelial cells and the sub-mucosa. (**B–D**) Immunostaining for intestinal GRP78 protein in the NEC group at 24 h, 48 h, and 72 h of NEC modeling shows GRP78-positive cells mainly located around the intestinal epithelial cells, mucosa and the sub-mucosa. (**E–G**) At 24 h, 48 h and 72 h of NEC modeling, the immunostaining of intestinal GRP78 protein in the FO + NEC group. FO pre-feeding reduced the expression of GRP78 protein in the intestinal mucosa.

On immunostaining, GRP78-positive cells exhibited yellow-brown staining in the cytoplasm and were mainly located around the intestinal mucosal epithelial cells (Fig. 5). The gray value analysis of GRP78 showed that the GRP78 protein expressions in the NEC group at 24 h, 48 h, and 72 h of NEC modeling were greater than those in the control group by 22.95%, 45.26%, and 38.62%, respectively (*P* < 0.05 at all 3 time-points) (Table 6). The GRP78 protein expressions in the FO + NEC group at 24 h, 48 h and 72 h of NEC modeling were greater than those in the control group by 14.59%, 29.85, and 27.15, respectively (P < 0.05 for all) and lesser than those in the NEC group by 6.80%, 10.61%, and 8.27%, respectively (P < 0.05 at all 3 time-points).

**Table 6:** Gray value analysis of intestinal GRP78 protein expression in rats (n = 8).

Group	0 h	24 h	48 h	72 h
Control	119.58 ± 5.82	120.14 ± 6.96	119.61 ± 6.02	120.51 ± 8.22
NEC	117.72 ± 7.50	147.71 ± 9.01^*Δ^	173.75 ± 11.88^*Δ^	167.05 ± 10.75^*^
FO + NEC	118.13 ± 5.90	137.67 ± 6.18^*#Δ^	155.31 ± 8.18^*#Δ^	153.23 ± 9.58^*#^

^*^P < 0.05 versus control group; ^#^*P* < 0.05 versus NEC group; ^△^*P* < 0.05 versus the immediately preceding time –point.

### Correlation between histopathological score and IL-6, TNF-α or caspase-12 in intestine of rats with NEC

In the NEC model, the expressions of GRP78 and caspase-12 protein in the intestine of rats showed a progressive increase over time during the process of the NEC (Table 7). Under NEC modeling, there was a rapid increase in caspase-12 protein at 24 h and it reached a peak at 72 h, while the GRP78 protein reached a peak at 48 h. The NEC group showed the greatest increase in the expressions of caspase-12 protein at 72 h of NEC modeling (77.12% greater than that in the control group) and GRP78 protein at 48 h of NEC modeling (45.26% greater than that in the control group). In addition, in the NEC group, we observed a strong positive correlation between intestinal histopathological score and the expression levels of IL-6, TNF-α, and caspase-12 [correlation coefficients at 24, 48, 72 hours: 0.914, 0.894, and 0.926, respectively (P < 0.05 for all), Table 7].

**Table 7:** Correlation of histopathological score with intestinal expressions of IL-6, TNF-α, and caspase-12 in rats.

Score	Sample amount	IL-6 (pg/mL)	TNF-α (pg/mL)	caspase-12 (gray value from Western blot)
≤1	64	152.14 ± 11.66	149.95 ± 10.50	0.5245 ± 0.03
1∼2	20	212.11 ± 22.86	196.67 ± 23.38	0.6976 ± 0.06
2∼3	9	254.44 ± 10.19	236.59 ± 12.53	0.8428 ± 0.07
3∼4	3	288.29 ± 4.50	265.51 ± 16.94	0.9929 ± 0.03
r		0.914	0.894	0.926
p		<0.001	<0.001	<0.001

## Discussion

It is well known that ERS and UPR activation are closely related to intestinal inflammation in mouse and human inflammatory bowel diseases^28^. In our study, the levels of ERS-related molecules (caspase-12 and GRP78) in the intestinal tissue increased during progression of NEC modeling. Furthermore, we observed a positive correlation of caspase-12 with IL-6 or TNF-α, which suggests that ERS is associated with intestinal inflammation and plays a crucial role in the pathogenesis of NEC. Our results are consistent with those of Lu *et al*.^10^ who reported increased expressions of GRP78 and inflammatory factors (IL-6 and IL-8) in intestinal tissue of children with NEC. In contrast, in our study, the intestinal pathological scores in the FO + NEC group were lesser than those in the NEC group by 38.79%. Further, during NEC modeling, the FO + NEC group showed decrease in caspase-12, GRP78, IL-6, and TNF-α by 20.80%, 10.61%, 13.70%, and 11.37%, respectively. Similarly, in a study by Zheng *et al*.^29^, DHA treatment significantly decreased the expression of GRP78 in mouse hepatocytes. These results indicate that the protective effect of FO on the intestinal tract of rats with NEC may be mediated via inhibition of inflammatory response which is associated with ERS.

In the present study, the intestinal pathological score in the NEC group showed a positive correlation with the levels of IL-6 and TNF-α. This result suggests that the level of intestinal pro-inflammatory cytokines may reflect the severity of intestinal mucosal damage in rats with NEC. In a study by Halpern *et al*^23^., treatment with anti-TNF-α reduced the incidence of NEC from 80% to 17%, which suggests that TNF-α plays an important role as a mediator in NEC progression. Pre-treatment of rats with FO in our study (FO + NEC group) remarkably reduced the level of IL-6 and TNF-α in rats with NEC at 48 h of modeling. In addition, the intestinal tissue pathological scores in the FO + NEC group were much lower than those in the NEC group. Consistent with these findings, ω-3 PUFAs were earlier shown to ameliorate postoperative intestinal inflammation and dysmotility^24^. DHA administration was shown to attenuate activation of inflammasomes induced by macrophage-derived TNF-α^25^. In patients with ulcerative colitis, EPA supplementation reduced mucosal inflammation, promoted differentiation of goblet cells, and modulated the intestinal microbiota composition^26^. In addition, IL-1 receptor associated kinase inhibitors were shown to reduce the levels of TNF-α, IL-1β, and IL-6, and further inhibit the inflammatory response in rats with NEC^27^. All these findings indicate a potential anti-NEC effect of FO.

In our study, we observed aggravation of NEC symptoms over time during NEC processing; at 72 h of NEC modeling, the phenomenon of intestinal luminal flatulence was observed at necropsy. However, mild intestinal luminal flatulence was observed in the group pretreated with FO. These results indicate that pre-treatment of rats with FO alleviated the symptoms of NEC. Similarly, administration of DHA and EPA was shown to reduce the incidence of colitis in an earlier study on rats^22^. In addition, insulin-like growth factor I intervention reduced the symptoms and the incidence rate of NEC in rats^6^. As DHA and EPA are two main ingredients of FO, we speculate that FO can be considered as a potential therapeutic agent for NEC.

In conclusion, FO pre-feeding of newborn rats attenuated NEC-induced intestinal mucosal damage and alleviated the accompanying clinical symptoms. Thus, FO may be considered as a single potential agent for alleviating the clinical symptoms of NEC. The protective effect of FO on the intestinal tract of rats with NEC may be mediated via inhibition of intestinal inflammatory response which is associated with ERS. However, the detailed signaling mechanisms that mediate the effect of FO on ERS require further study.

## Data Availability

The data supporting the results of the present investigation are available from the corresponding author upon reasonable request.
